# Neutralizing antibody response to different COVID-19 vaccines in Brazil: the impact of previous infection and booster doses

**DOI:** 10.3389/fimmu.2025.1603612

**Published:** 2025-08-04

**Authors:** Beatriz L. L. Caetano, Paolla B. A. Pinto, Agatha R. Pacheco, Agnes R. Lage, Aline S. G. Pereira, Amanda V. P. Nascimento, Thiago R. Machado, Anderson Paulino, Thiago L. Medeiros, Lorena O. Fernandes-Siqueira, Andrea T. Da Poian, Ingrid S. Horbach, Adriana S. Azevedo, Simone M. Costa, Ada M. B. Alves

**Affiliations:** ^1^ Laboatório de Biotecnologia e Fisiologia de Infecções Virais, Instituto Oswaldo Cruz, Fundação Oswaldo Cruz, Rio de Janeiro, RJ, Brazil; ^2^ Instituto de Bioquímica Médica Leopoldo de Meis, Universidade Federal do Rio de Janeiro, Rio de Janeiro, RJ, Brazil; ^3^ Laboratório de Análise Imunomolecular, Instituto de Tecnologia em Imunobiológicos, Bio-Maguinhos, Fundação Oswaldo Cruz, Rio de Janeiro, RJ, Brazil

**Keywords:** COVID-19, vaccine, neutralizing antibodies, booster dose, SARS-CoV-2

## Abstract

**Introduction:**

In Brazil, three COVID-19 vaccines were among the first widely used (CoronaVac, ChAdOx1, and BNT162b2), which aimed to induce neutralizing antibodies (NAbs) against the original SARS-CoV-2 strain. Although effective against severe disease, they showed waning NAb levels and reduced efficacy against variants, prompting booster doses. Thus, it is important to investigate and compare the response induced by these vaccines and boosters.

**Methods:**

In this study, we compare the magnitude, durability, and cross-reactivity of NAbs among vaccinated volunteers in Brazil using an enzyme-linked immunosorbent assay (ELISA)-based assay that measures Abs capable of blocking the interaction between the receptor binding domain (RBD) and human angiotensin-converting enzyme 2 (ACE2) receptor.

**Results:**

The BNT162b2 two-dose regimen resulted in the highest and most durable NAb levels, followed by ChAdOx1, while those induced by CoronaVac significantly declined over time. Breakthrough infections boosted NAb levels, especially for CoronaVac and ChAdOx1. All vaccines showed reduced neutralizing capacity against Gamma, Delta, and Omicron variants. Booster doses, particularly the first one, significantly increased and maintained NAb levels, including those against Omicron.

**Discussion:**

Our findings provide valuable population-based comparison of NAb levels elicited by different vaccines following primary inoculation and booster doses. Notably, the mRNA vaccine exhibited a strong primary and initial booster NAb response against SARS-CoV-2.

## 1 Introduction

In Brazil, three COVID-19 vaccines were initially widely used following approval by the National Health Surveillance Agency (ANVISA): CoronaVac, which was composed of the inactivated SARS-CoV-2 developed by the Sinovac Biotech Company (1) and produced by the Brazilian Butantan Institute; ChAdOx1 nCoV-19, which was based on an adenoviral vector developed by the Oxford University together with AstraZeneca Pharmaceutical (2) and produced in Brazil by the Oswaldo Cruz Foundation; and the mRNA-based BNT162b2 vaccine, developed by the BioNTech Company together with Pfizer Pharmaceutical (3). All these vaccines target the spike protein of the original SARS-CoV-2 strain as their main antigen aiming to reach a neutralizing antibody (NAb)-centered immunity. Unlike the other two platforms, CoronaVac may additionally induce antibodies against other structural viral proteins, although the spike protein remains the primary target for neutralization. Phase 3 clinical trials showed effectiveness against COVID-19, with CoronaVac at 50.7%–83.5% (4–6), ChAdOx1 at 70.4% (7), and BNT162b2 at 94%–95% (3). All three vaccines have shown seroconversion with neutralizing activity against SARS-CoV-2. However, follow-up studies revealed a decline in SARS-CoV-2 vaccine-induced antibody levels over time, along with lower vaccine efficacy against the constantly emerging SARS-CoV-2 viral variants (8–11). As a result, booster vaccinations using either the original or updated COVID-19 vaccines were recommended and remain in place today.

Although controlled clinical studies have confirmed the efficacy and safety of COVID-19 vaccines, direct comparisons are challenging due to variations in different populations and regions of the world, vaccination timing, and the prevalence of emerging viral variants. Population-based data offer a valuable and comprehensive source of tracking vaccination outcomes and should be considered when formulating future COVID-19 vaccination strategies (12).

The present study compares the magnitude, durability, and cross-reactivity of the NAb response among volunteers in Brazil who were vaccinated first with two doses of CoronaVac, ChAdOx1, or BNT162b2, followed by homologous or heterologous booster doses. The two-dose standard vaccination with BNT162b2 induced the strongest and most sustained responses, while CoronaVac showed the fastest decline. Prior infection enhanced responses across all groups. Boosters—especially the first—were essential to restore and maintain antibody levels, improve protection against variants like Omicron, and support long-term immunity up to 1 year post-vaccination. This study provides valuable insights into the dynamics and duration of vaccine-induced antibody responses, which are crucial for informing future guidelines on vaccine dosing regimens and heterologous dose combinations in combating the pandemic.

## 2 Materials and methods

### 2.1 Study population

The study included healthy volunteers residing in Rio de Janeiro, Brazil, who had received one or two doses of routine COVID-19 vaccination either with CoronaVac (Sinovac/Butantan), ChAdOx1 (Oxford/AstraZeneca), or BNT162b2 (Pfizer/BioNTech) and who were subsequently administered heterologous or homologous booster doses. Participants in the study were required to be over 18 years of age, sign an informed consent form, and provide detailed demographic information, including sex and date of birth, as well as data on previous SARS-CoV-2 infections identified through rapid tests and polymerase chain reaction (PCR). The study protocol was approved by the Ethics Committee of Oswaldo Cruz Institute (CEP-IOC) - Fiocruz (license numbers: CAAE 51345021.5.1001.5248 and CAAE 56246022.1.0000.5248) and of the Federal University of Rio de Janeiro (license number: 35.303.120.5.0000.5257).

A total of 506 individuals voluntarily participated in the study. The age distribution was categorized into two groups: 18 to 59 years old (*n* = 425), comprising 307 women and 118 men, and 60 years or older (*n* = 81), comprising 57 women and 24 men. Determination of previous SARS-CoV-2 infection was based on participants’ self-reported positive results from PCR and/or rapid tests, as well as specific seroconversion to the viral nucleocapsid protein in the enzyme-linked immunosorbent assay (ELISA).

### 2.2 Sample collection and study design

Blood samples were collected by venipuncture in vacuum tubes (BD Vacutainer, BD Bioscience) containing sodium heparin anticoagulant by trained personnel. Each volunteer donated two tubes with 9 mL of blood. Plasma was obtained after centrifugation of heparinized blood at 1,000 × *g* for 10 min at room temperature (R.T.), aliquoted, and stored at −80°C until use.

Participants were vaccinated according to Brazil’s national immunization campaign, which began in January 2021, with prioritization based on age, comorbidities, and occupational exposure. The sample collection occurred between mid-2021 and late 2023, depending on volunteer availability, and was not restricted to specific variant of concern (VOC) waves or vaccine batches. There was no active control over participants’ vaccination timing, beyond their self-reported vaccination status and willingness to participate in this study. It is worth mentioning that between early 2021 and 2023, Brazil experienced distinct SARS-CoV-2 waves driven by variants like Gamma (early 2021), Delta (mid- to late 2021), and Omicron (December 2021 through 2023 with sub-lineages), each significantly impacting public health.

The study design included the collection of plasma samples after each dose of the COVID-19 vaccine at different time intervals. Groups were stratified according to their initial vaccination regimen of first and second homologous doses with either CoronaVac, ChAdOx1, or BNT162b2. Sample collections after the third and fourth vaccine doses were adopted as subsequent heterologous or homologous booster doses. In Brazil, the CoronaVac vaccine fell into disuse during the COVID-19 booster vaccination, leading to a preference for heterologous booster regimens involving either the ChAdOx1nCoV-19 or BNT162b2 vaccines. The sample collection timing intervals varied by each vaccine-recommended protocol and volunteer willingness and availability. Sample times were designated as follows: T1, average time according to each vaccine stipulated regimen, varying from 15 to 86 days; T2, 15 to 75 days after the second dose; T3, 90 to 365 days after the second dose; T4, 15 to 75 days after the third dose; T5, 90 to 365 days after the third dose; T6, 15 to 75 days after the fourth dose; and T7, 90 to 365 days after the fourth dose. The average time of T1 collection varied according to the vaccine due to different vaccination regimens in Brazil: 23 days for CoronaVac, 71 days for ChAdOx1, and 66 days for BNT162b2. We clarify that the data presented in this study are predominantly treated as a cross-sectional cohort, with statistical treatment of individual samples as independent observations. While a limited subset of participants provided samples at multiple time points, it was not possible to monitor and collect samples from all participants at all designated time points throughout the study period (withdrawal from vaccination or participation in the study).

### 2.3 Quantification of SARS-CoV-2-specific neutralizing activity

Detection of NAb was performed using the cPass™ SARS-CoV-2 Neutralization Antibody kit (GenScript, cat# L00847), according to the manufacturer’s instructions. The assay utilizes a recombinant receptor binding domain (RBD) of the SARS-CoV-2 spike protein. It quantifies Abs that block the interaction between the RBD and the human angiotensin-converting enzyme 2 (ACE2) receptor. The cPASS assay has received regulatory validation from the U.S. Food and Drug Administration as a reliable tool for SARS-CoV-2 neutralization, since, as stated by the agency document, “the test mimics the virus neutralization process” (13).

Briefly, plasma samples along with positive and negative controls provided with the kit were diluted in the sample dilution buffer and incubated with RBD conjugated to horseradish peroxidase (RBD-HRP) for 30 min at 37°C. Following incubation, the reaction mixtures were transferred to microplates pre-coated with ACE2 protein for 15 min at 37°C. The RBD-HRP bound to ACE2 was detected using tetramethylbenzidine (TMB, Sigma) substrate for 15 min at R.T. followed by a stopping solution. Optical density (O.D.) was measured at 450 nm using a GloMax Explorer GM3500 microplate reader (Promega). Plasma samples were incubated in single replicates, while controls were incubated in duplicates.

The NAb data were expressed in percentage or concentration in international units/mL (IU/mL).

For the percentage representation, the binding inhibition was calculated as follows:


$$\text{Inhibition }\left(\%\right)\;=(1-(\frac{\text{sample O}.\text{D}.\text{ value}}{\text{negative control O}.\text{D}.\text{ value}})\;\times 100)$$

As standardized by the manufacturer, the ≥30% cutoff was adopted for interpretation of positive SARS-CoV-2 neutralization activity. An inhibition percentage of ≥30% indicates the presence of Abs interacting with SARS-CoV-2 RBD and blocking the RBD–hACE2 interaction. The percentage of inhibition in plasma samples was assessed against original SARS-CoV-2 RBD (Wuhan isolate) as well as against its variants Gamma (GenScript, cat# Z03601), Delta (GenScript, cat# Z03614), and Omicron BA.1 (GenScript, cat# Z03730).

For the concentration representation as IU/mL, a semiquantitative analysis was also conducted, especially those collected after the third and fourth vaccine doses, using commercially provided SARS-CoV-2 Neutralizing Antibody Standard curves (GenScript, cat# A02087). This methodological shift was necessary due to the higher antibody levels, which approached the percentage detection threshold. Results were also expressed as antibody concentrations in IU, facilitating comparison with other assays that quantify SARS-CoV-2 NAbs (14).

### 2.4 Quantification of neutralizing antibodies against SARS-CoV-2 using classical PRNT

The Plaque Reduction Neutralization Test (PRNT), considered the gold standard for assessing NAbs against various viruses, including SARS-CoV-2, was also used in this study. Its results were compared with the cPass™ SARS-CoV-2 Neutralization Antibody kit as a way to validate our findings on viral neutralization inhibition. This comparison was performed with a total of 114 plasma samples, including CoronaVac (*n* = 38), ChAdOx1 (*n* = 37), and BNT162b2 (*n* = 40).

The PRNT protocol was previously described in detail (15). In summary, Vero cells (CCL81, ATCC) were seeded into 24-well plates (2 × 10^5^ cells/well) in 199 media with Earle salts (E199, Sigma) supplemented with 5% fetal bovine serum (FBS, Invitrogen) 1 day before the assay. Plasma samples were heat-inactivated at 56°C for 30 min, serially diluted in culture medium (1:10 to 1:31,250), and incubated with approximately 60 plaque-forming units (PFU) of the ancestral strain of SARS-CoV-2 (SISGEN A994A37—donation from the Laboratory of Respiratory Viruses, Exanthematics, Enteroviruses and Viral Emergencies at IOC/Fiocruz) for 1 h at 37°C in a 5% CO_2_ atmosphere. The plasma–virus mixture was added to Vero cell monolayers and incubated for 1 h at 37°C in 5% CO_2_. After this incubation, the supernatants were discarded, and cells were covered with 199 media supplemented with 5% FBS and 1.5% carboxymethylcellulose (CMC, Sigma), incubated for 3 days at 37°C in 5% CO_2_, followed by fixation and inactivation with 1.25% (v/v) formalin solution and stained with 0.04% (w/v) crystal violet dye. Plaques were counted manually. Finally, NAb titers were expressed as the highest serum dilution that resulted in 50% plaque reduction (PRNT50), considering samples with titers ≥ 1:14 seropositive to SARS-CoV-2. This threshold was established based on a receiver operating characteristic (ROC) curve analysis, which used 46 negative and 378 positive samples. The analysis identified 1.64 log_5_ as the optimal cutoff, corresponding to a dilution of 1:14. This point achieved the best balance between sensitivity and specificity, maximizing the assay’s discriminatory power between positive and negative samples. PRNT assays were handled in a BSL-3 laboratory Multi-user Research Facility of Biosafety Platform BSL3-HPP, Oswaldo Cruz Institute, Oswaldo Cruz Foundation, Rio de Janeiro, Brazil, following the approved international laboratory biosafety guidelines (CDC, Interim Laboratory Biosafety Guidelines for Handling and Processing Specimens Associated with Coronavirus Disease 2019).

### 2.5 Quantification of SARS-CoV-2-specific N antibody response

Plasma samples from all time points were evaluated for specific nucleocapsid (N) seroconversion as an indicator of previous SARS-CoV-2 infections. We quantified specific IgG Abs against the N protein from the original SARS-CoV-2 strain (Wuhan isolate) using a previously developed and validated ELISA protocol (16).

In summary, 96-well plates (Corning) were coated with the recombinant N protein (0.2 μg/mL) produced in human embryonic kidney (HEK) 293 cells and incubated overnight at 4°C. The following day, plates were washed with PBST [0.1% Tween in phosphate-buffered saline (PBS)] and blocked with 3% bovine serum albumin (BSA) in PBST for 1 h at R.T. The blocking solution was removed, and samples diluted 50-fold were added to the plates and incubated for 2 h at room temperature. After washing, the plates were incubated with secondary antibody (anti-IgG-HRP) diluted 1:5,000 in PBST for 1 h at 4°C. Plates were washed again and reactions were developed with TMB (50 μL per well) for 16 min at room temperature and then stopped with 3M HCl (50 μL per well). Absorbances were read at 450 nm on a spectrophotometer (Glomax Discover, Promega).

In this study, 15 samples collected prior to the COVID-19 pandemic (from individuals who had never been exposed to SARS-CoV-2) were used to determine the seroconversion positivity threshold for the N protein. The mean O.D. values plus three times the standard deviation was calculated and set as the cutoff for positivity. A pool of these samples was used as a control on all ELISA plates. Volunteers’ samples with O.D. higher than the stablished threshold were considered positive for previous SARS-CoV-2 infection.

### 2.6 Statistics

Results were statistically analyzed using GraphPad Prism software, version 9.0 (La Jolla, USA). All graphical data are presented as the median and interquartile range (IQR). Statistical differences were assessed using the non-parametric Mann–Whitney test for two groups; non-parametric Kruskal–Wallis test with *post hoc* Dunn’s correction for multiple comparisons was used for more than two groups. Correlation analyses were performed by computing Spearman’s rank correlation coefficient and significance in GraphPad Prism. A *p*-value< 0.05 was considered statistically significant. Statistical comparisons involving very small sample sizes (*n*< 5) should be interpreted with caution.

## 3 Results

### 3.1 Study population characteristics

The study cohort comprises 452 volunteers initially vaccinated with two homologous doses of CoronaVac, ChAdOx1, or BNT162b2, including a total of 321 female and 131 male participants. Participants were categorized by age: 354 individuals were between 18 and 59 years old (272 women and 82 men), and 98 were aged 60 and above (49 women and 49 men).

Among those vaccinated with CoronaVac (*n* = 108), there were 81 women (75%) and 27 men (24%), with median ages of 47 and 45 years, respectively. In this group, 59 women (54.6%) and 18 men (16.6%) were aged 18 to 59 years, while 22 women (20.4%) and 9 men (8.4%) were ≥60 years. The ChAdOx1 group (*n* = 285) was the most representative cohort, consisting of 206 women (72.2%) and 79 men (27.8%), with median ages of 40 and 47 years, respectively. Within this group, 180 female (63.2%) and 40 male participants (14%) were aged 18 to 60 years, and 26 women (9.1%) and 39 men (13.7%) were 60 years or older. The BNT162b2 vaccine was used later in Brazil; thus, this group is smaller and with young volunteers, with 33 women (55.9%) and 24 men (40.7%), with median ages of 28 and 31 years, respectively. Of these, 33 women (55.9%) and 24 men (40.7%) were aged 18 to 59 years, and 1 woman (1.7%) and 1 man (1.7%) were ≥60 years (**Table 1**). The impact of sex and age groups was assessed during the data analysis in this study.

**Table 1:** Demographic data for the COVID-19 vaccinated volunteers.

	All volunteers (*n* = 452)		CoronaVac (*n* = 108)		ChAdOx1 (*n* = 285)		BNT162b2 (*n* = 59)	
	Female	Male	Female	Male	Female	Male	Female	Male
**Total,** ***n* (%)**	321 (71.0)	131 (29.0)	81 (75.0)	27 (25.0)	206 (72.2)	79 (27.8)	34 (57.6)	25 (42.3)
**Median age ** **(range)**	40 (15-92)	44 (18-84)	47 (19-86)	45 (21-62)	40 (20-83)	47 (19-84)	28 (18-61)	31 (18-60)
***n* (%) of Age group: 18 to 59**	272 (60.2)	82 (18.2)	59 (54.6)	18 (16.6)	180 (63.2)	40 (14.0)	33 (55.9)	24 (40.7)
***n* (%) of Age group: ≥ 60**	49 (10.8)	49 (10.8)	22 (20.4)	9 (8.4)	26 (9.1)	39 (13.7)	1 (1.7)	1 (1.7)

The demographic data for the volunteer cohort were stratified based on the initial vaccination regimen, which included two homologous doses from the CoronaVac, ChAdOx1, and BNT162B2 vaccine groups, followed by sex and age of each group. Age is represented in years.

The distribution of the total samples for each vaccine group collected at the different time points (T1 to T7) is detailed in **Table 2**. Specifically, 183 samples were collected from volunteers vaccinated with CoronaVac, 630 samples were collected from volunteers vaccinated with ChAdOx1, and 129 samples were collected from volunteers vaccinated with BNT162b2, totaling 942 samples at different times throughout the study (**Table 2** **, top**). The distribution of samples across time points was not uniform because participant recruitment occurred continuously throughout the study period. The increased number of participants at T3 is likely attributable to a rise in public interest regarding vaccine effectiveness, together with the relaxation of social distancing measures. In general, participation in the study decreased over time as the COVID-19 pandemic progressed and booster doses were administered, affecting the number of samples of all vaccine groups.

**Table 2:** Total number of collected samples by time point.

	All samples (*n* = 937)	CoronaVac (*n* =183)	ChAdOx1 (*n* = 626)	BNT162b2 (*n* = 128)
Total of collected samples, *n* (%)				
T1	197 (21.0)	22 (12.0)	141 (22.5)	34 (26.6)
T2	149 (15.9)	26 (14.2)	94 (15.0)	29 (22.7)
T3	217 (23.2)	65 (35.5)	127 (20.3)	25 (19.5)
T4	112 (12.0)	25 (13.7)	73 (11.7)	14 (10.9)
T5	149 (15.9)	30 (16.4)	108 (17.3)	11 (8.6)
T6	31 (3.3)	4 (2.2)	24 (3.8)	3 (2.3)
T7	82 (8.7)	11 (6.0)	59 (9.4)	12 (9.4)
*^#^Previous SARS-CoV-2 infection, *n* (%)				
T1	27 (13.7)	3 (13.6)	20 (14.2)	4 (11.8)
T2	34 (22.8)	10 (38.5)	18 (19.1)	6 (20.7)
T3	39 (18.0)	16 (24.6)	18 (14.2)	5 (20.0)
T4	39 (34.8)	5 (20.0)	27 (37.0)	7 (50.0)
T5	73 (49.0)	17 (56.7)	52 (48.1)	4 (36.4)
T6	12 (38.7)	1 (25.0)	9 (37.5)	2 (66.7)
T7	51 (62.2)	7 (63.6)	38 (64.4)	6 (50.0)

*Previous SARS-CoV-2 infection was determined through self-reported information from volunteers up to 6 months before sample collection, and/or by detecting seropositivity for the viral N protein.

^#^The numbers and percentages shown represent the proportion of positive samples out of the total samples collected at each time point.

Collection time intervals: T1—average time according to each vaccine stipulated regimen, varying from 15 to 86 days; T2—15 to 75 days after the second dose; T3—90 to 365 days after the second dose; T4—15 to 75 days after the third dose; T5—90 to 365 days after the third dose; T6—15 to 75 days after the fourth dose; T7—90 to 365 days after the fourth dose.

Previous SARS-CoV-2 infection was determined either by self-reported positive results from PCR and/or rapid tests from volunteers up to 6 months prior to sample collection or by specific seroconversion to the viral nucleocapsid protein. The number of samples considered positive for previous SARS-CoV-2 infection is detailed in **Table 2** (bottom). The impact of prior infection on the vaccine-induced antibody response was evaluated in the study.

### 3.2 Variation in the SARS-CoV-2 NAb response induced by CoronaVac, ChAdOx1, and BNT162b2 vaccination

**Figure 1:**
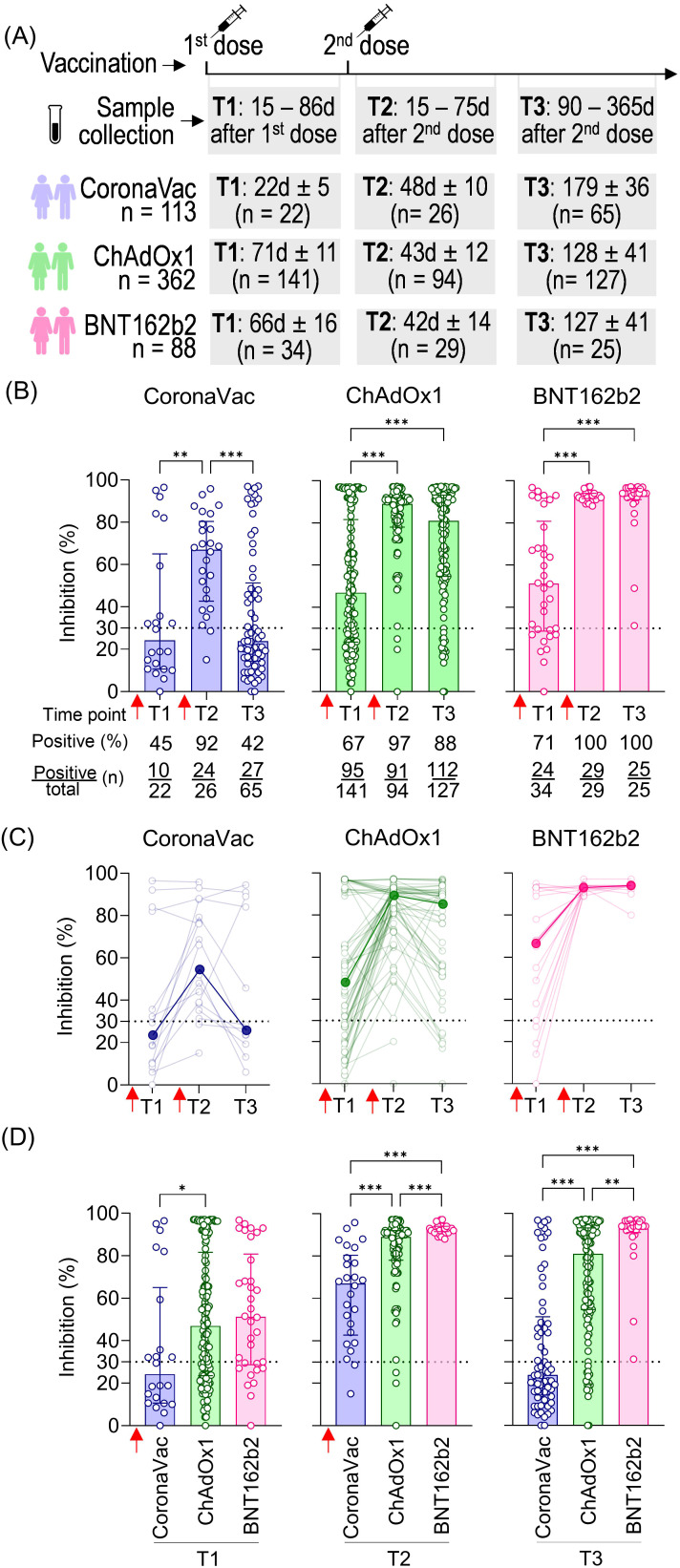
SARS-CoV-2 NAb response induced by two homologous doses of CoronaVac, ChAdOx1, and BNT162b2 vaccination. **(A)** Timeline of sample collection of vaccinated volunteers who received standard two-dose vaccine regimens with CoronaVac, ChAdOx1, or BNT162b2. Plasma samples were collected at the following time points: T1—15 to 86 days after the first dose; T2—15 to 75 days after the second dose; T3—90 to 365 days after the second dose. The average time for T1 varied according to the recommended schedule for each vaccine, being shorter for CoronaVac (average of 23 days) and longer for ChAdOx1 (average of 71 days) or BNT162b2 (average of 66 days). The number of volunteers per group and the average time of collection ± standard deviation are detailed. **(B)** Percentage of NAb in individuals vaccinated with one or two homologous doses of CoronaVac, ChAdOx1, or BNT162b2 vaccines. **(C)** Longitudinal follow-up of vaccinated volunteers with at least two subsequent collections through time points. Medians for each time point were connected by a bold line to better represent the results. **(D)** Comparison of NAb percentages at each time point according to the three different vaccine regimens. **(B–D)** Specific NAb percentages were assessed using the cPass™ SARS-CoV-2 Neutralization Antibody with the RBD from the original SARS-CoV-2 strain (Wuhan isolate). The dashed line at 30% indicates the test positivity threshold. Red arrows indicate vaccine doses. The bars indicate the median and IQR. Non-parametric Kruskal–Wallis test was used for statistical analyses. *p< 0.05, **p< 0.01, ***p< 0.001. Sample sizes of **(C)**—CoronaVac: T1 (*n* = 14), T2 (*n* = 19), and T3 (*n* = 13); ChAdOx1: T1 (*n* = 62), T2 (*n* = 75), and T3 (*n* = 49); BNT162b2: T1 (*n* = 19), T2 (*n* = 22), and T3 (*n* = 10). Samples sizes of **(B, D)** are detailed in **(A)**.

The dynamics of NAb induced by vaccination were evaluated in samples from volunteers who received two homologous doses of CoronaVac, ChAdOx1, or BNT162b2 at three time points: collection after the first dose (T1: average time according to each vaccine stipulated regimen, varying from 19 to 84 days), short-term collection after the second dose (T2: 15 to 75 days), and long-term collection after the second dose (T3: 90 to 365 days). The average time of T1 collection varied according to the vaccine due to different vaccination regimens in Brazil: 23 days for CoronaVac, 71 days for ChAdOx1, and 66 days for BNT162b2. The total number of volunteers per group and the average time intervals for each time point are summarized in **Figure 1A**.

The NAb response against SARS-CoV-2 was measured using the cPass™ SARS-CoV-2 Neutralization Antibody ELISA kit (GenScript). Results were expressed as percentage inhibition, with 30% considered the positive threshold. CoronaVac induced the lowest NAb response (median: 24%), followed by ChAdOx1 (median: 47%) and BNT162b2 (median: 51%) after the first dose (T1), with the respective seroconversion rates of 45%, 67%, and 71%. All vaccinated groups showed a significant increase in the percentage of NAb shortly after the second dose (T2), with 67% for CoronaVac, 89% for ChAdOx1, and 93% for BNT162b2. The second dose was crucial (T2), also evidenced by the high seroconversion rates 48 days after vaccination with CoronaVac (92%; 24 out of 26 individuals), 43 days after vaccination with ChAdOx1 (97%; 91 out of 94 individuals), and 42 days after vaccination with BNT162b2 (100%; 29 out of 29 individuals). Antibody persistence was assessed at T3 (90 to 365 days after the second dose). Individuals vaccinated with CoronaVac showed a significant decline in neutralizing capacity in T3 compared to T2, approximately 179 days after the second dose, with an average of 24% of NAb and 27 out of 65 individuals considered positive for seroconversion. ChAdOx1 and BNT162b2 groups showed a slight decline in NAb levels, with an average of 129 and 127 days after the second dose of each, although no statistically significant difference was observed comparing T2 and T3. Both groups maintained high seroconversion rates of 88% (112 out of 127 individuals) and 100% (25 out of 25 individuals), respectively (**Figure 1B**).

Longitudinal analysis of ChAdOx1 and CoronaVac vaccinees showed an initial increase in NAb levels shortly after the second dose (T2) followed by a decline long after the second dose (T3). However, BNT162b2 consistently maintained high and uniform NAb levels (**Figure 1C**). Comparative analysis of NAb levels shortly (T2) and long (T3) after the second dose demonstrated significant differences between vaccine strategies, with BNT162b2 showing a more homogeneous and higher NAb response compared to the other two. Notably, the standard two-dose CoronaVac regimen consistently induced lower percentages of NAb than ChAdOx1 and BNT162b2 at the two time points evaluated (T2 and T3) (**Figure 1D**). Our findings indicate that the standard two-dose regimen induces varying NAb responses against the original SARS-CoV-2 virus. CoronaVac elicited lower and less durable NAb, while ChAdOx1 produced a robust but also less durable response. In contrast, BNT162b2 generated a significantly stronger NAb response, characterized by higher levels, greater persistence, and overall robustness.

To validate the use of the cPass SARS-CoV-2 Neutralization Antibody ELISA kit with our cohort, we performed the gold standard plaque reduction neutralization titer assay (PRNT50) using plasma samples previously characterized as low, medium, and high neutralizing. A significant correlation was observed between the two assays, with a Spearman’s rank correlation coefficient of *r* = 0.74 (*p*< 0.0001) (**Supplementary Figure S1A**). Individually, the correlations were as follows: *r* = 0.72 (*p*< 0.0001) for CoronaVac (**Supplementary Figure S1B**), *r* = 0.73 (*p*< 0.0001) for ChAdOx1 (**Supplementary Figure S1C**), and *r* = 0.69 (*p*< 0.0001) for BNT162B2 (**Supplementary Figure S1D**).

### 3.3 Previous infection with SARS-CoV-2 impacts the vaccine-induced NAb response after CoronaVac, ChAdOx1, and BNT162b2 vaccination

To assess the impact of previous SARS-CoV-2 infection on the vaccine-induced NAb response, we considered self-reported information from volunteers up to 6 months before the sample collection and/or detection of seropositivity for the viral N protein.

**Figure 2:**
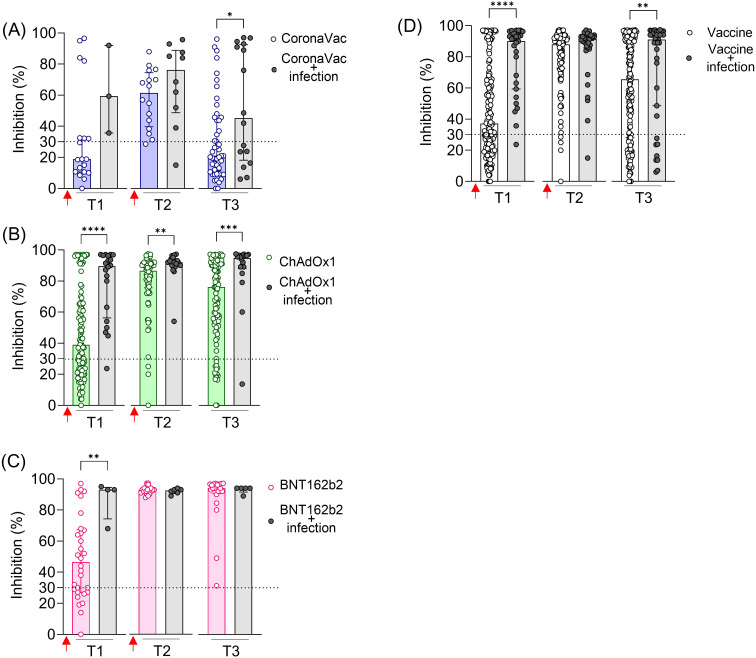
SARS-CoV-2 NAb in individuals vaccinated with CoronaVac, ChAdOx1, or BNT162b2, with and without previous SARS-CoV-2 infection. Percentage (%) of NAb in individuals vaccinated with the standard two-dose regimen with CoronaVac **(A)**, ChAdOx1 **(B)**, BNT162b2 **(C)**, and all vaccines together **(D)**. Specific NAb percentages were assessed using the cPass™ SARS-CoV-2 Neutralization Antibody with the RBD from the original SARS-CoV-2 strain (Wuhan isolate). Previous SARS-CoV-2 infection was determined by self-reported positive results from PCR and/or rapid tests, as well as specific seroconversion to the viral nucleocapsid protein. The dashed line at 30% indicates the test positivity threshold. Red arrows represent vaccine doses. The bars indicate the median and IQR. Non-parametric Mann–Whitney test was used for statistical analyses. *p< 0.05; **p< 0.01; ***p< 0.001; ****p< 0.0001. Sample sizes: T1—CoronaVac (*n* = 19/3), ChAdOx1 (*n* = 121/20), and BNT162b2 (*n* = 30/4); T2—CoronaVac (*n* = 16/10), ChAdOx1 (*n* = 76/18), and BNT162b2 (*n* = 23/6); T3—CoronaVac (*n* = 49/16), ChAdOx1 (*n* = 109/18), and BNT162b2 (*n* = 20/5); values indicate number of vaccine-only/vaccine + infection participants.

Previous SARS-CoV-2 infection significantly increased NAb levels against the original SARS-CoV-2 strain (Wuhan isolate) after the first dose (T1) in all vaccinated groups (**Figure 2**). In the CoronaVac and ChAdOx1 groups, individuals with previous infection exhibited higher average NAb levels shortly after the second dose (T2) and, more notably, at a later time point (T3), when these differences were statistically significant for both vaccines (**Figures 2A, B**). In contrast, individuals vaccinated with BNT162b2, regardless of having a previous SARS-CoV-2 infection, achieved high levels of NAb shortly and long after the second dose (T2 and T3), approaching the assay’s limit of detection (**Figure 2C**). Because of the limited number of samples at certain time points in the vaccinated and infected groups, we combined the results from the three vaccinated groups (CoronaVac, ChAdOx1, and BNT162b2) and assessed the impact of prior infection, irrespective of the vaccination regimen. The average NAb values were higher in the group with prior SARS-CoV-2 exposure, both after the first dose (T1) and long-term follow-up after the second dose (T3) (**Figure 2D**).

These results indicate that prior infection with SARS-CoV-2 enhanced the specific NAb induced by two-dose vaccinations with CoronaVac and ChAdOx1, including the durability of these neutralizing responses. However, this prior infection had a minimal impact on the BNT162b2 regimen.

### 3.4 Dynamic comparison of NAb induced by CoronaVac, ChAdOx1, or BNT162b2 vaccination and their respective booster doses in individuals with and without previous SARS-CoV-2 infection

**Figure 3:**
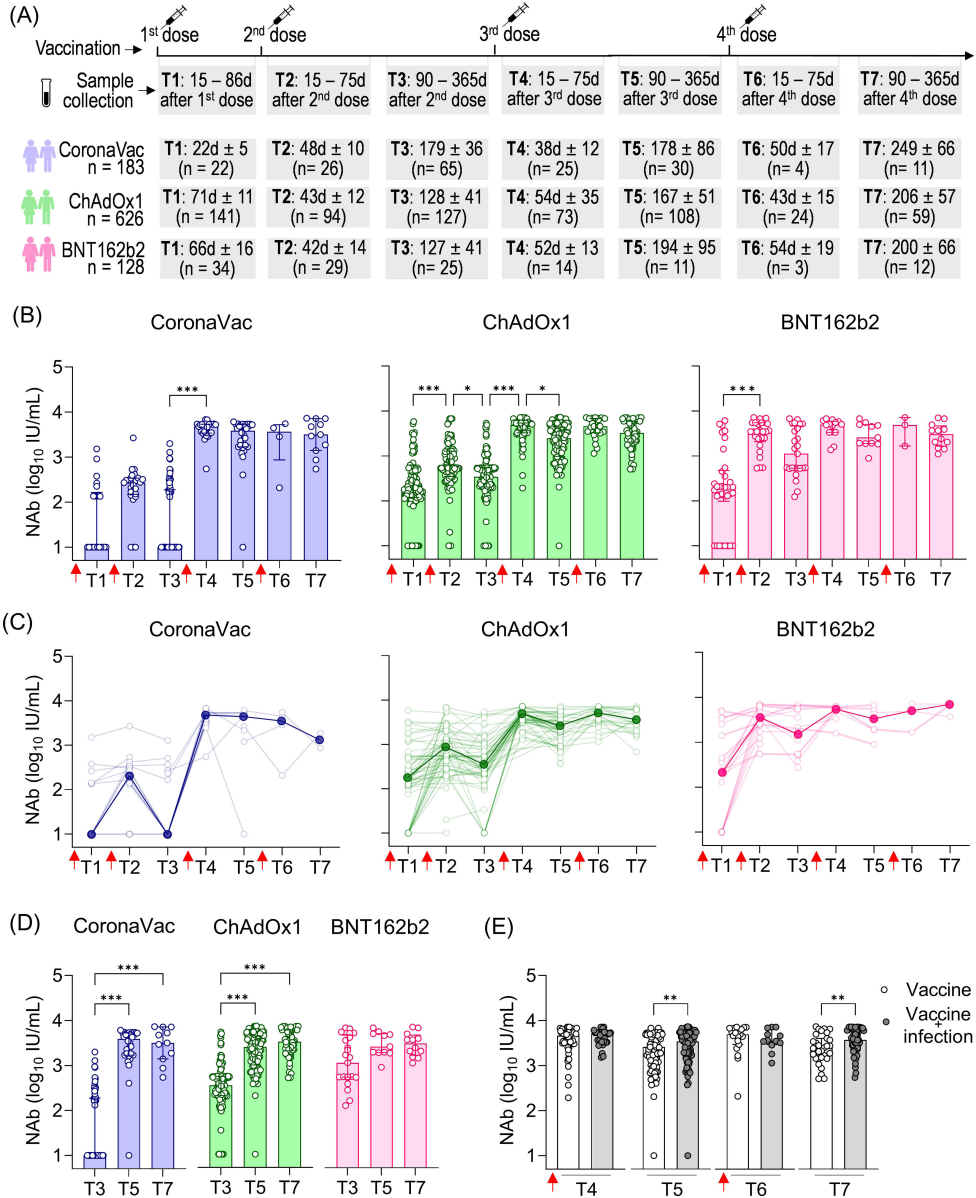
SARS-CoV-2 NAb response induced by two homologous doses of CoronaVac, ChAdOx1, and BNT162b2 vaccination followed by booster doses. **(A)** Timeline of sample collection of vaccinated volunteers who received the first standard two-dose vaccine regimen with CoronaVac, ChAdOx1, or BNT162b2 vaccines followed by the booster doses. Plasma samples were collected at the following time points: T1—15 to 86 days after the first dose; T2—15 to 75 days after the second dose; T3—90 to 365 days after the second dose. The average time for T1 varied according to the recommended schedule for each vaccine, being shorter for CoronaVac (average of 23 days) and longer for ChAdOx1 (average of 71 days) or BNT162b2 (average of 66 days). The number of volunteers per group and the average time of collection ± standard deviation are detailed. **(B)** Concentration (IU/mL) of NAb against the original SARS-CoV-2 strain (Wuhan isolate) in volunteers who received the initial vaccine regimen of CoronaVac, ChAdOx1, or BNT162b2, followed by one or two booster doses of any of these vaccines. **(C)** Longitudinal follow-up of the NAb concentration (IU/mL) for participants with two or more consecutive sample collections from T1 to T7. Medians for each time point were connected by a bold line to better represent the results. **(D)** Concentration (IU/mL) of NAb against the original SARS-CoV-2 strain (Wuhan isolate) in vaccinees at long-term time points (T3, T5, and T7). Concentrations (IU/mL) of NAb were assessed using the cPass™ SARS-CoV-2 Neutralization Antibody with the RBD from the original SARS-CoV-2 strain (Wuhan isolate) and a standard monoclonal antibody curve (GenScript). **(E)** Concentration of NAb in vaccinated individuals (CoronaVac, ChAdOx1, and BNT162b2) followed by booster doses with or without previous SARS-CoV-2 infection. **(B–D)** The bars indicate the median and IQR. Red arrows indicate vaccine doses. **(B, D)** Non-parametric Kruskal–Wallis test was used for statistical analyses. *p< 0.05, ***p< 0.001. **(E)** Non-parametric Mann–Whitney test was used for statistical analyses. **p< 0.01. Sample sizes of **(C)**—CoronaVac: T1 (*n* = 20), T2 (*n* = 23), T3 (*n* = 15), T4 (*n* = 8), T5 (*n* = 6), T6 (*n* = 3), and T7 (*n* = 3); ChAdOx1: T1 (*n* = 70), T2 (*n* = 75), T3 (*n* = 70), T4 (*n* = 56), T5 (*n* = 54), T6 (*n* = 25), and T7 (*n* = 23); BNT162b2: T1 (*n* = 14), T2 (*n* = 19), T3 (*n* = 29), T4 (*n* = 20), T5 (*n* = 10), T6 (*n* = 4), and T7 (*n* = 2). Sample sizes of **(E)**—Vaccine only: T4 (*n* = 73), T5 (*n* = 76), T6 (*n* = 19), and T7 (*n* = 31); Vaccine + infection: T4 (*n* = 39), T5 (*n* = 73), T6 (*n* = 12), and T7 (*n* = 51). Samples sizes of **(B, D)** are detailed in **(A)**.

As the COVID-19 pandemic evolved with the emergence of new variants, booster vaccine dose regimens were implemented. To better quantify high-level NAb responses that may exceed the limits of percentage-based measurements following booster doses, we expressed NAb levels in international units per milliliter (IU/mL), calculated by using a commercially SARS-CoV-2 NAb standard curve. Samples were collected at various time points following the recommended vaccination schedule: after the first dose (T1), 15 to 75 days after the second dose (T2), 90 to 365 days after the second dose (T3), 15 to 75 days after the third dose (T4), 90 to 365 days after the third dose (T5), 15 to 75 days after the fourth dose (T6), and 90 to 365 days after the fourth dose (T7). The total number of volunteers per group and the average time intervals for each time point are summarized in **Figure 3A**. In Brazil, heterologous booster doses (using a different vaccine platform than the initial regimen) were prioritized. Moreover, CoronaVac was less commonly used for boosters. Most booster doses for ChAdOx1 recipients were BNT162b2, and contrariwise. For the CoronaVac group, both ChAdOx1 and BNT162b2 were administered as boosters.

The first booster dose (third vaccine dose) was crucial in increasing NAb levels shortly after that dose, particularly in individuals who received CoronaVac (479.7-fold increase) or ChAdOx1 (14.1-fold increase) as the initial vaccine regimen, where T4 was statistically higher than T3. Although not statistically significant in the BNT162b2 group, NAb levels showed a 4.4-fold increase shortly after the first booster dose (third vaccine dose) between T3 and T4. The short-term impact of the second booster dose (fourth vaccine dose) seems less prominent regarding the NAb response against the original virus, as there was no significant statistical increase in values observed at T6 (shortly after the fourth dose) compared to the values observed both shortly and long after the third dose (T3 and T4, respectively) (**Figure 3B**). Longitudinal analysis of vaccinees showed a similar pattern of responses, with a prominent increase in NAb levels shortly after the first booster dose, especially for CoronaVac and ChAdOx1, followed by high and uniform NAb levels (**Figure 3C**).

The long-term persistence of NAb following vaccination is evident in the extended collection times, ranging from 90 to 365 days after each dose: T3 (after the second dose), T5 (after the third dose), and T7 (after the fourth dose) (**Figure 3D**). Comparatively, we observe much lower NAb levels long after the second dose (T3) compared to long after the third dose (T5) in the CoronaVac and ChAdOx1 vaccinees, underscoring the importance of the first booster dose in enhancing the durability of NAb responses against SARS-CoV-2 regarding these vaccines. In the CoronaVac group, NAb levels were approximately 386.1-fold lower in T3 (average of 179 days after the second dose) compared to T5 (average of 178 days after the third dose). However, the fourth dose resulted in a non-significant small decrease comparing NAb in T5 (average of 178 days after the third dose T5) and T7 (average of 249 days after the fourth dose). Regarding the ChAdOx1 vaccine, NAb levels were statistically 7.3-fold lower in T3 (average of 128 days after the second dose) compared to T5 (average of 167 days after the third dose), with a less pronounced increase (1.3-fold) in T7 (average of 206 days after the fourth dose) compared to T5. In contrast, individuals who initially received the BNT162b2 regimen maintained consistently high and long-lasting NAb levels, with no significant difference observed between T3 (average of 127 days after the second dose), T5 (average of194 days after the third dose), and T7 (average of 200 days after the fourth dose) (**Figure 3D**).

We assessed the influence of sex and age on the NAb response. Owing to the limited number of individuals in some subgroups, we combined all vaccinated individuals at different time points and also separated them by the first vaccine regimens (the first two homologous doses) (**Supplementary Figure S2**). The influence of sex was only evident shortly after the second booster dose (T6), where male individuals had higher NAb levels than female individuals (**Supplementary Figure S2A**). Regarding age, we observed that individuals aged 60 years or older had lower NAb levels only at the longest time point after vaccination (T3), but this difference was not seen in either shortly (T4 and T6) or long (T5 and T7) after booster doses (**Supplementary Figure S2A**).

To assess the influence of prior SARS-CoV-2 infection on NAb dynamics after booster doses, we analyzed data from all vaccinated individuals together (CoronaVac, ChAdOx1, and BNT162b2). While no significant impact of prior infection was observed in the shorter follow-up times after the third (T4) and fourth (T6) vaccine doses, we found statistically higher NAb levels in vaccinated individuals and individuals previously infected with SARS-CoV-2 at the longer time points (T5 and T7) (**Figure 3E**). This suggests that prior infection mainly affects the durability of NAb responses rather than their magnitude.

Taken together, these results highlight the importance of booster doses in enhancing the antibody response to the original SARS-CoV-2 strain. The first booster played a significant role in increasing the NAb levels both shortly and long after vaccination, while the second booster was slightly more relevant in promoting greater durability of these antibodies (Abs), which was also correlated with the impact of prior SARS-CoV-2 infection.

### 3.5 Cross-reactivity of NAb response induced by CoronaVac, ChAdOx1, or BNT162b2 vaccination directed to Gamma, Delta, and Omicron variants

A major difficulty in COVID-19 vaccination is promoting a robust and durable cross-protective immune response against emerging SARS-CoV-2 VOCs leading to ongoing infection waves and prolonging the pandemic. To assess antibody cross-reactivity, we examined their neutralization capacity against Gamma (P.1), Delta (B.1.617.2), and Omicron (B.1.1.529) VOCs.

**Figure 4:**
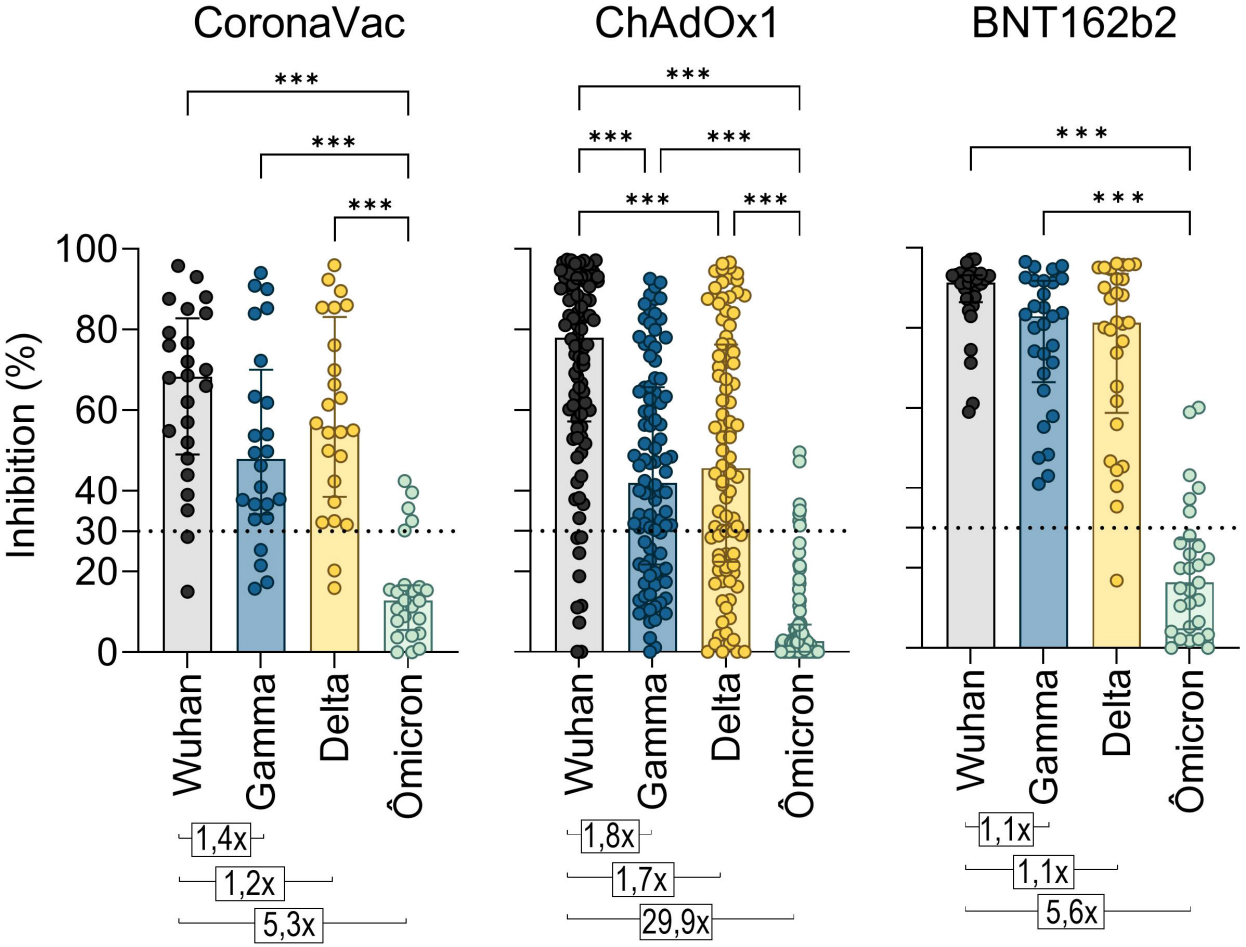
Neutralizing antibody response to SARS-CoV-2 Gamma, Delta, and Omicron variants induced by two homologous doses of CoronaVac, ChAdOx1, and BNT162B2 vaccination. Percentage (%) of NAb in individuals shortly after vaccination with CoronaVac, ChAdOx1, or BNT162b2, against the original virus RBD or its VOCs Gamma, Delta, and Omicron. The bars represent the median and IQR. Different plasma dilutions were used for samples of each vaccination regimens: CoronaVac, 10-fold; AstraZeneca, 50-fold; Pfizer, 100-fold. The dashed line at 30% represents the positivity detection threshold of the test. Values above the bars represent the average ratios between the NAb percentage against the original virus RBD and against the RBDs of the Gamma, Delta, and Alpha VOCs. The Kruskal–Wallis non-parametric test was used for statistical analysis. ***p< 0.001. Sample sizes—CoronaVac (*n* = 24), ChAdOx1 (*n* = 94), and BNT162b2 (*n* = 29).

A standard vaccination regimen with two homologous doses of CoronaVac, ChAdOx1, or BNT162b2 showed a greater capacity to neutralize the original SARS-CoV-2 strain compared to the Gamma, Delta, and Omicron variants shortly after vaccination (T2) (**Figure 4**). The NAb median response against the Gamma and Delta variants was above the positive threshold for all three vaccines, though it remained lower compared to the original strain. For Gamma, the NAb percentages were approximately 47.9% for CoronaVac, 41.9% for ChAdOx1, and 82.9% for BNT162b2 groups. Against Delta, the averages were 55.9%, 45.6%, and 81.4%, respectively. However, none of the regimens demonstrated effective neutralization against the highly mutated Omicron variant, which exhibited the lowest average NAb levels (CoronaVac: 12.8%, ChAdOx1: 2.6%, and BNT162b2: 16.3%) and the largest reduction ratio relative to the original strain (CoronaVac: 5.3-fold, ChAdOx1: 29.9-fold, and BNT162b2: 5.6-fold). Comparatively, CoronaVac and BNT162b2 groups showed the lowest rates of antibody reduction against VOCs, while the ChAdOx1 vaccinees exhibited the highest reduction (**Figure 4**).

**Figure 5:**
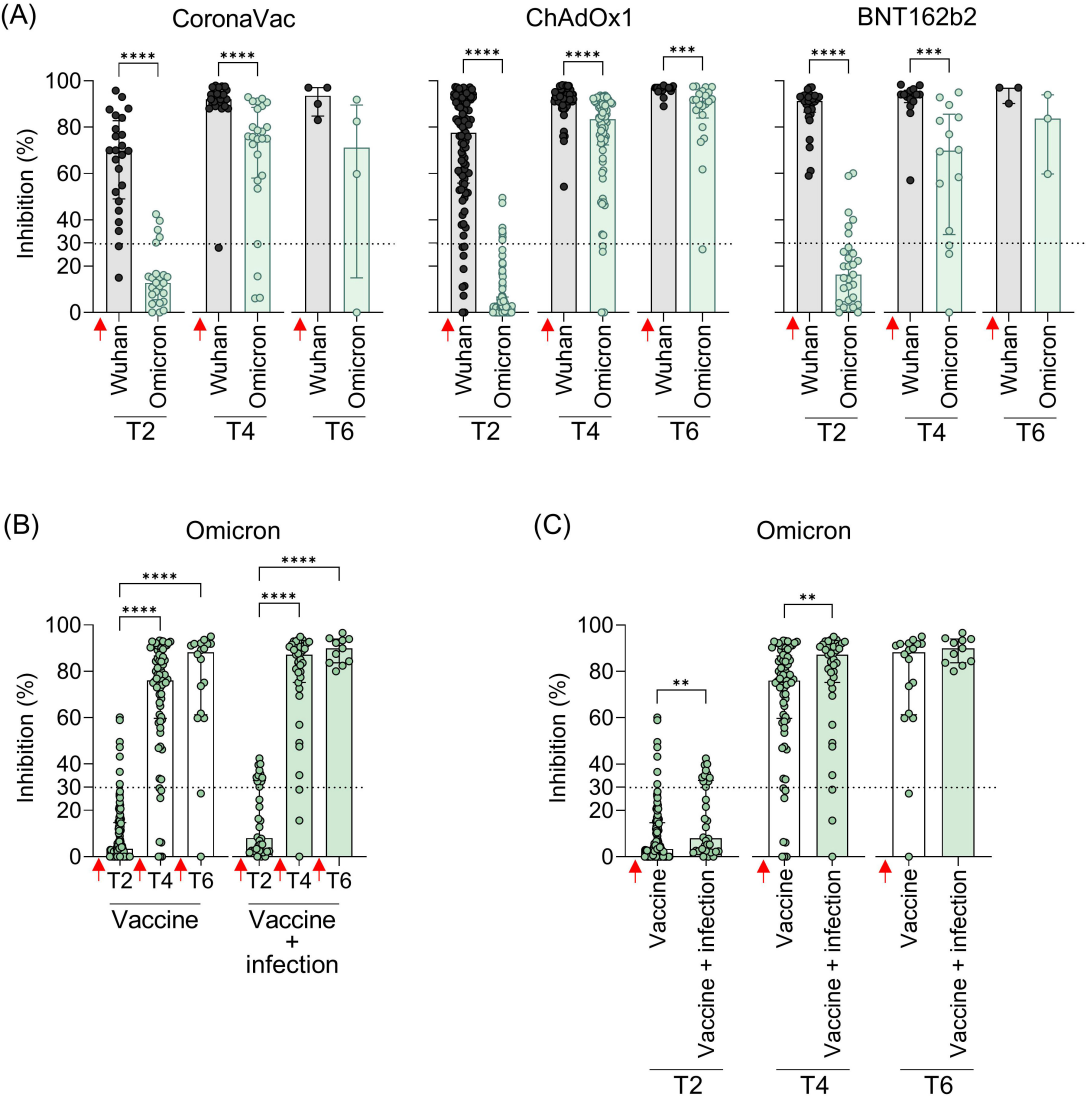
Neutralizing antibodies against the original virus and the Omicron VOC in individuals vaccinated with CoronaVac, ChAdOx1, or BNT162b2 followed by booster doses, with and without previous SARS-COV-2 infection. **(A)** Percentage (%) of NAb against the original SARS-CoV-2 virus and Omicron variant at the different time points. **(B, C)** Percentage (%) of NAb against the Omicron variant in vaccinated individuals with and without previous virus infection. Plasma samples were collected at the following time points: T2—15 to 75 days after the second dose; T4—15 to 75 days after the third dose; T6—15 to 75 days after the fourth dose. Specific NAb percentages were assessed using the cPass™ SARS-CoV-2 Neutralization Antibody with the RBD from the original SARS-CoV-2 strain (Wuhan isolate) or Omicron (B.1.529.1). Previous SARS-CoV-2 infection was determined by self-reported positive results from PCR and/or rapid tests, as well as specific seroconversion to the viral nucleocapsid protein. The bars represent the median and IQR. Red arrows indicate vaccine doses. Non-parametric Mann–Whitney test was used for statistical analyses. **p< 0.01; ***p< 0.001; **** *p*< 0.0001. Sample size of **(A)**—CoronaVac: T2 (*n* = 24), T4 (*n* = 25), and T6 (*n* = 4); ChAdOx1: T2 (*n* = 94), T4 (*n* = 73), and T6 (*n* = 22); BNT162b2: T2 (*n* = 29), T4 (*n* = 14), and T6 (*n* = 3). Sample size of **(B, C)**—Vaccine only: T2 (*n* = 113), T4 (*n* = 73), and T6 (*n* = 18); Vaccine + infection: T2 (*n* = 34), T4 (*n* = 39), and T6 (*n* = 11).

Given the widespread prevalence of Omicron since late 2021, the cross-reactivity of Ab induced by booster doses was evaluated exclusively against this variant shortly after vaccination. Statistically, all vaccine regimens showed lower levels of NAb against Omicron compared to the original strain, both after the second (T2) and third doses (T4). While a reduction in NAb levels was observed after the fourth dose, it was statistically significant only in the ChAdOx1 group (**Figure 5A**). Over time (T2 to T6), NAb levels showed a tendency to increase. To investigate whether this rise was driven solely by the booster doses or also by natural infections with ongoing Omicron waves, samples from all vaccine groups were analyzed, distinguishing between vaccination alone and vaccination with SARS-CoV-2 infection before collection. A clear increase in NAb levels against both the original strain and Omicron was observed after booster doses compared to the initial response from the homologous vaccine regimen (**Figure 5B**). Antibody levels against Omicron were higher in individuals who had both been vaccinated and infected with SARS-CoV-2, with statistically significant differences at T2 and T4 (**Figure 5C**). After the second dose of the homologous regimen (T2), SARS-CoV-2 infection increased the Omicron NAb response, even though the Omicron variant was not circulating when these samples were collected. This increase was more evident after the third (T4) dose, with NAb levels rising from 76% to 87.2% (**Figure 5C**).

## 4 Discussion

The unprecedented development of COVID-19 vaccines has been crucial in combating the pandemic. The ongoing accumulation of population-based vaccination data provides valuable insights to understand how to optimize vaccine use in the future. Although not exclusively, the development of COVID-19 vaccines prioritized inducing a NAb response, primarily targeting the highly antigenic S protein, which is crucial for viral entry. Several studies with individuals vaccinated against COVID-19 demonstrate a positive correlation between high levels of NAb and vaccine protective efficacy (17–19). In Brazil, the population was primarily vaccinated with three COVID-19 vaccines: CoronaVac (inactivated virus), ChAdOx1 (viral vector), and BNT162b2 (mRNA). Since different vaccine platforms can induce varying immune responses, this study aimed to evaluate the magnitude and durability of NAb up to 365 days after the vaccination following booster doses and its ability to neutralize viral variants.

While PRNT50 is the gold standard for measuring NAb against SARS-CoV-2 (20) and other viruses, its complexity limits its use (costly, time-consuming, and the need for a BSL3 facility). As an alternative, this study used a commercially available Food and Drug Administration (FDA)-approved immunoenzymatic assay (cPass Neutralizing Antibody kit) that measures ACE2-RBD binding inhibition as a surrogate for NAb levels (13, 21). Previous studies correlate well such an assay with PRNT results (22–26), which was also seen here with a subset of our vaccinated cohort.

Our initial data show that the two-dose homologous regimen of the BNT162b2 vaccine generated higher and long-lasting NAb levels against SARS-CoV-2, followed by ChAdOx1 and finally CoronaVac. Direct comparisons of our findings with the literature are challenging, as COVID-19 vaccination worldwide involved not only these vaccines but also several others, administered in varying orders and schedules. Similar results were seen comparing these three different vaccine platforms (27, 28). Healthcare workers vaccinated with mRNA vaccines (mRNA-1273 and BNT162b2) exhibited significantly higher NAb titers after the first dose, and these levels remained elevated 6 months after the second dose, compared to those vaccinated with ChAdOx1 or Sinopharm (28). Similar patterns have been reported in Mexico (29), Thailand (30), Chile (31), Indonesia (32), and Brazil (33, 34), where studies consistently showed that BNT162b2 induced superior NAb responses compared to other vaccine platforms. Other studies have shown that vaccine-induced SARS-CoV-2 NAb tends to decrease over time (8–10, 35), affecting the long-lasting protective immunity against this virus. One multicentric study conducted in Brazil and Mexico has shown that BNT162b2 offers a more sustained SARS-CoV-2 Spike IgG response in a 6-month follow up (36). Our results, in addition to others (36–38), revealed that CoronaVac induced lower and less durable levels of NAbs than BNT162b2 and ChAdOx1, indicating that the platform of the inactivated virus is less effective. Furthermore, the immunogenic epitopes of the spike protein may undergo structural changes during the virus inactivation process, potentially affecting and reducing its immunogenicity (39). However, it is important to note that the CoronaVac and its platform was of extreme importance at the beginning of the pandemic in Brazil, by inducing protection against severe COVID-19 and deaths, being applied to health workers and the elderly (40), as well as in other countries around the world (4–6). The mRNA vaccines, in turn, mimic natural infection, leading to high-affinity antibody production and prolonged antigen protein production, which sustained the immune response (41).

Initially, vaccines were administered in a single vaccination schedule with two homologous doses, followed by booster doses in response to the observed decline in antibody neutralization over time and the ongoing evolution of viral variants capable of evading vaccine-induced immunity. In Brazil, boosters were primarily BNT162b2 for the CoronaVac and ChAdOx1 groups, and mainly ChAdOx1 for the BNT162b2 group. The first booster dose (third vaccination) significantly strengthened and sustained the NAb response over time, especially for CoronaVac and ChAdOx1 initial regimens. Booster doses of COVID-19 vaccines, particularly with heterologous regimens, have been shown to enhance antibody responses, including against emerging variants responsible for new infection waves (42–45). According to our findings, the mRNA vaccines (BNT162b2 and mRNA-1273) have been especially effective in reinforcing immunity initially induced by inactivated or viral vector vaccines (46–48). Studies from Brazil (49), Chile (50), and Thailand (51) show a robust increase in vaccine-induced NAb response when the BNT162b2 vaccine was administered as booster for individuals initially vaccinated with CoronaVac. These findings are important for designing future vaccine guidelines in low- and middle-income countries that relied on CoronaVac for their vaccination campaigns.

Several factors can alter the production and durability of NAb generated by COVID-19 vaccines, such as age, sex, and previous infections (52). In general, vaccines have lower efficacy in older individuals due to age-related immunosenescence (53, 54). However, our results showed similar immunogenicity regardless of age, in accordance with other vaccination studies (55, 56). Elderly individuals showed lower NAb levels in long-term follow-up after the initial vaccination regimen, suggesting an age-related impact in sustaining the vaccine’s immunogenicity, but this difference was no longer apparent after booster doses. A single difference emerged after the booster dose, with men demonstrating higher NAb titers. This sex-specific difference, though not widely observed, has been reported previously (57) and may be related to factors not investigated here such as hormonal factors, genetic variations, or testosterone’s impact on immune response activation (58).

The challenge of evaluating COVID-19 vaccine immunogenicity is that vaccination has occurred alongside waves of breakthrough infections likely caused by VOCs with high capacity to evade vaccine-induced immunity (59). Therefore, assessing the NAb response against these VOCs and the impact of natural infection on vaccine immunity is essential. Our findings demonstrate that regardless of the initial vaccine (CoronaVac, ChAdOx1, or BNT162b2), the Gamma, Delta, and especially Omicron variants showed reduction in NAb recognition. The reduced recognition of NAb against SARS-CoV-2 VOCs has been demonstrated for all vaccines, highlighting mutations mainly in the RBD as potent mediators of immune escape from the vaccine response (60–62). Even with NAb evasion, individuals infected with Omicron, which presents substantial differences compared to the original virus (63), typically experience mild disease (64), suggesting the involvement of other immune mechanisms, such as the T-cell response, in disease control and modulation.

One of the major benefits of administering booster doses against COVID-19 was the increase in protection against SARS-CoV-2 VOCs (65, 66). We observed an increase in NAb against Omicron booster doses, which may be associated with the stimulation of the immune system following antigen re-exposure, either from natural infection or from an additional vaccine booster dose. We showed that individuals previously infected with SARS-CoV-2 exhibited higher NAb levels after COVID-19 vaccination and that this prior infection had a significant impact on the long-term sustainability of the antibody response after booster doses, according to other studies (29, 31, 33, 67–69). Our data support that not only the effect of booster doses was responsible for increasing vaccine-induced immunity against the Omicron VOC, but also natural breakthrough infections occurring concomitantly with the immunization period, even when another VOC was circulating. These findings align with previous studies showing higher NAb production, including an enhanced ability to recognize VOCs (70, 71), probably due to an amplification of vaccine-induced memory immune response either by hybrid immunity from natural infection or by booster vaccination. In line with our findings where the majority of vaccinees received BNT162b2 as a heterologous booster dose, the use of mRNA vaccines has been of important value in strengthening immunity against SARS-CoV-2 VOCs (72–74).

This study has limitations. First, sample sizes were uneven across time points and participant subgroups, with relatively fewer elderly individuals and a higher proportion of women than men. Similarly, the representation of previously infected individuals varied across time points. Sample size for stratification based on the different vaccines used for booster doses (ChAdOx1 or BNT162b2) was also insufficient to clearly determine whether the results observed were associated with the vaccine platforms or even the impact of homologous boosters. Second, we were unable to follow all participants longitudinally, which constrained our ability to assess individual-level antibody dynamics over time. Third, we did not assess comorbidities, medication use, or other medical factors, as this information was not available for all volunteers, preventing us from establishing their potential impact on the NAb vaccine-induced response. Finally, the study focused exclusively on humoral immune responses, without evaluating cellular immunity, which plays a crucial role in vaccine-induced protection and long-term immune memory. Our population-based data indicate that the two-dose mRNA BNT162b2 vaccine generated stronger and more durable NAb responses compared with inactivated and vector-based vaccines. The booster doses, particularly the third vaccination, were essential, especially for CoronaVac and ChAdOx1, significantly increasing NAb levels, including against VOCs like Omicron, which was previously undetectable. This enhanced response was attributed to booster doses and/or natural infection. The mRNA platform proved more effective in generating a stronger and more durable NAb response and played a highly effective role as a booster vaccine.

## Data Availability

The data of this study can be provided but not the confidential information about the participants. Requests to access the datasets should be directed to ada@ioc.fiocruz.br.
