# Supplementary material for: Neutralizing antibody response to different COVID-19 vaccines in Brazil: the impact of previous infection and booster doses
Source: Front Immunol. 2025 Aug 4;16:1603612. doi: 10.3389/fimmu.2025.1603612 (PMC12359843; doi:10.3389/fimmu.2025.1603612)
Supplement: Supplementary file 1 [file DataSheet1.docx]

Supplementary Material


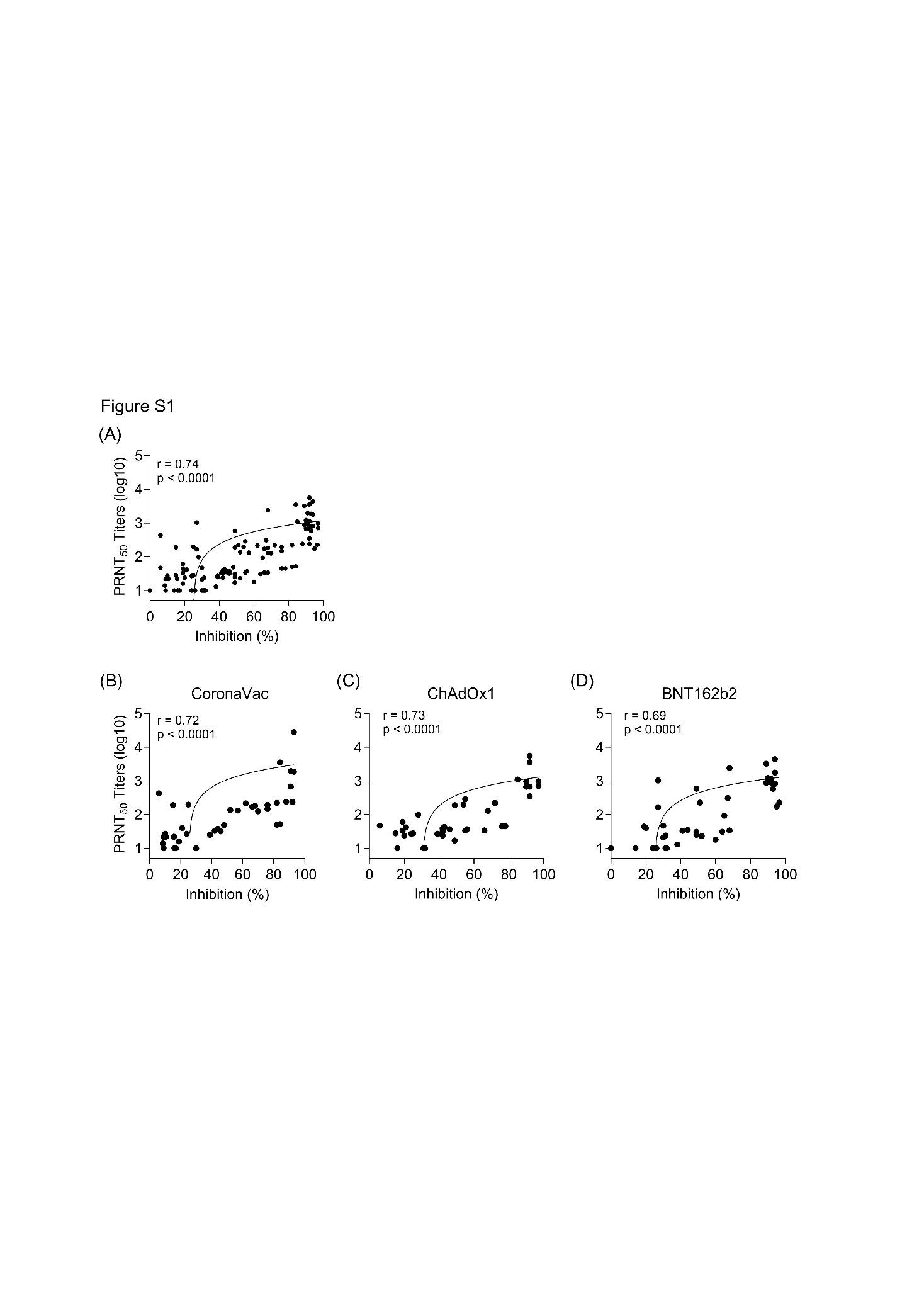


**Figure S1.** Correlation of cPass™ SARS-CoV-2 Neutralization Antibody ELISA kit and PRNT50 for detecting SARS-CoV-2 neutralizing antibodies against SARS-CoV-2. Plasma samples from volunteers vaccinated with two homologous doses of **(A)** CoronaVac (n = 38), **(B)** ChAdOx1 (n = 37), or **(C)** BNT162b2 (n = 40) collected at T2 were analyzed using the cPass™ SARS-CoV-2 Neutralization Antibody and PRNT50. The r and p values for the correlations were determined by two-tailed Spearman’s correlations.


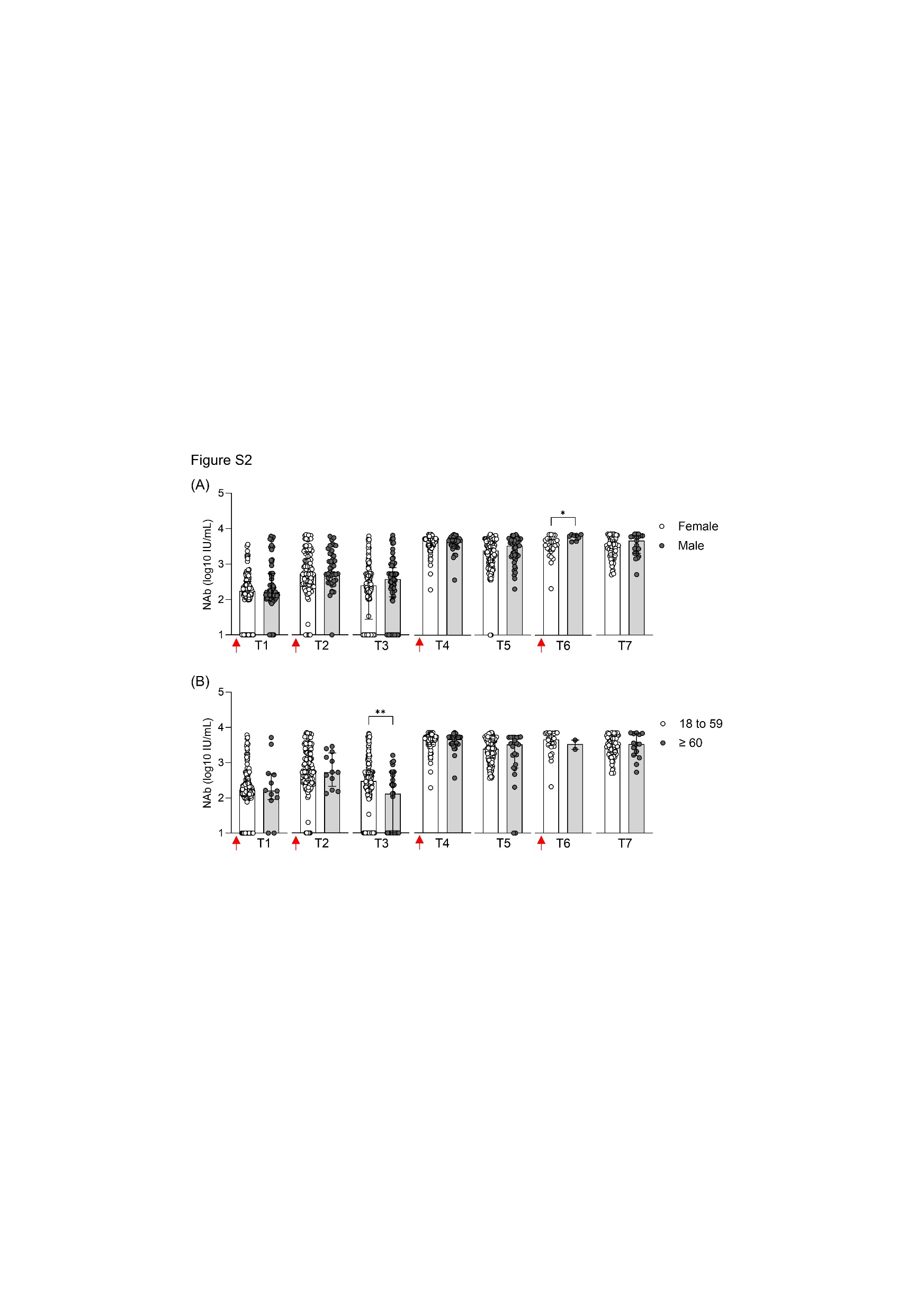


**Figure S2: Influence of sex and age in the NAb induced by two homologous doses of CoronaVac, ChAdOx1, and BNT162b2 vaccination followed by booster doses.** Concentration (IU/mL) of NAb in vaccinated individuals (CoronaVac, ChAdOx1, and BNT162b2) followed by booster doses separated by sex (male and female) **(A)** or age groups (18 to 59 years and 60 years or older) **(B)**. The bars indicate the median and IQR. Red arrows indicate vaccine doses. Non-parametric Mann-Whitney test was used for statistical analyses. *p < 0.05; **p < 0.01. Sample sizes – Female: T1 (n=142), T2 (n=105), T3 (n=154), T4 (n=80), T5 (n=102), T6 (n=23), T7 (61); Male: T1 (n=58), T2 (n=45), T3 (n=64), T4 (n=32), T5 (n=47), T6 (n=8), T7 (21); 18 to 59 years: T1 (n=185), T2 (n=137), T3 (n=182), T4 (n=83), T5 (n=126), T6 (n=29), T7 (68); ≥ 60 years: T1 (n=12), T2 (n=12), T3 (n=35), T4 (n=29), T5 (n=23), T6 (n=2), T7 (14).
